# Edaphic Selection Pressures as Drivers of Contrasting White Spruce Ectomycorrhizal Fungal Community Structure and Diversity in the Canadian Boreal Forest of Abitibi-Témiscamingue Region

**DOI:** 10.1371/journal.pone.0166420

**Published:** 2016-11-11

**Authors:** Martin B. Nadeau, Damase P. Khasa

**Affiliations:** Centre for Forest Research, Institute of Integrative and Systems Biology, and Canadian Research Chair in Forest and Environmental Genomics, Université Laval, Quebec city, QC, Canada, G1V0A6; Friedrich Schiller University, GERMANY

## Abstract

Little is known about edaphic selection pressures as drivers of contrasting white spruce ectomycorrhizal fungal community structure and diversity in the Canadian boreal forest. We hypothesized that community composition differs among the four sites sampled–nursery, mining site, forest edge, and natural forest. Ectomycorrhizal (ECM) fungal community structure and diversity was studied at the four locations with soil fertility gradient through morpho-molecular and phylogenetic analyses in relationships with rhizospheric soil chemical properties. 41 different species were identified. Mining site had a significantly different species composition than the surrounding environments. Soil pH and percentage of roots colonized by ECM fungi increased while soil P, N, Fe, C, K, Mg, Al, Ca, and Na contents declined across the soil fertility gradient: nursery → natural forest → forest edge → mining site. Contrary to the preference of acid soils by ECM fungi, a few ecologically adapted to high pH, poor soil chemical fertility, and low organic matter content colonize white spruce roots on the non-acidogenic mining site, allowing natural regeneration of white spruce seedlings. Other ECM fungi are adapted to high fertigation level of commercial nursery. This study clearly shows the contrasting difference in white spruce ectomycorrhizal fungal community structure and diversity driven by edaphic selection pressures.

## Introduction

Ectomycorrhizal (ECM) fungi have been an important component of temperate forest ecosystems since they first evolved more than 200 million years ago [1]. In the boreal forest, they are symbiotically associated with fine roots of conifer tree species such as white spruce (*Picea glauca* [Moench] Voss), black spruce (*Picea mariana* [Mill.] BSP), jack pine (*Pinus banksiana* Lamb.) and balsam fir (*Abies balsamea* [L.] Mill.), but also with fine roots of various deciduous species such as trembling aspen (*Populus tremuloides* Michx.), green alder (*Alnus viridis*), speckled alder (*Alnus incana* [L.) Moench), white birch (*Betula papyrifera* Marsh.), and willow (*Salix* spp.) [2,3,4,5,6,7,8,9,10]. These symbiotic microorganisms supply nutrient elements and water to plants in exchange for carbohydrates that are essential for their development [11,12,13,14]. It has been reported that ECM fungi can utilise important soil elements (P, N, Mg, Ca, K, Zn, Cu, Ni, S, Mn, B, and Fe), which are generally unavailable to plants as organic and insoluble forms, by producing specific organic acids and enzymes [15,16,17,18]. As a result, ECM fungi play a vital role in tree nutrition, especially in low fertility soils of the boreal forest where most soil elements are stored in mineral rocks and organic matter [16,19,20]. In contrast to arbuscular mycorrhizal fungi, which have mostly colonised plant roots in nutrient-rich soils with near-neutral pH, ECM fungi generally favour soils with low pH and low mineralization rates [21].

Boreal forests are well known for having high ECM fungal species diversity [4,5,22,23,24]. Like plants, ECM fungal communities go through succession over time until forest ecosystems reach climax stage [25]. Species diversity decreases considerably following disturbance events, which return forest stands to early-successional stages [13]. At the beginning, the ecosystem is colonised by pioneer species that are adapted to site conditions [25]. Early successional forest stands usually have low ECM fungal species richness and are mainly composed of generalists [13]. In late-successional stages, both generalist and specialist species are present in forest stands and diversity is greatly augmented due to increasing site and soil complexity [13]. ECM fungi also generally display high genetic diversity within species [26]. This high genetic diversity generates intraspecific physiological variation among strains [26,27,28,29]. Mycelial growth, plant colonisation, enzyme production, plant growth stimulation, pH and temperature optimum for mycelial growth, tolerance to harsh conditions such as heavy metal toxicity and drought, nutrient uptake, and organic acid secretion by ECM fungi may differ greatly among strains of the same species [14,26,27,28,30]. Therefore, some species and strains may be better adapted than others to abiotic stressors.

ECM fungal species diversity and composition considerably influence the growth and nutrient uptake of host trees, thereby affecting ecosystem productivity [31,32]. ECM diversity at the local scale is controlled by disturbance, nutrient partitioning, and competition and other interactions with other microorganisms [33]. Many abiotic and biotic factors such as soil chemistry, microclimate and stand age are known to play an important role in determining the distributions of ECM fungal species and the composition of their communities [34,35]. Moreover, ECM fungal community structure in the boreal forest has often been strongly correlated with soil properties such as extractable ammonium, base saturation, and soil pH [21,36].

White spruce is one of the most commercially valuable, important, and widespread tree species of the Canadian boreal forest [6,37]. We have little knowledge regarding the structure of ECM fungal communities that are associated with white spruce across a soil fertility gradient of different ecosystems and habitats, and how abiotic stress can shape ECM fungal communities and diversity in different niches. In a recent study on influences of soil environment and willow host species on ectomycorrhizal fungi communities across a strong environmental gradient, Erlandson *et al*. [38] found that fungal abiotic niche determine the fungal species available to associate with host plants within a habitat. Therefore, studying the structure and diversity of the white spruce ECM fungal community across a strong environmental gradient is a key first step towards understanding symbiotic modulation that occurs both temporally and spatially, a primary influence on ECM community structure, thereby allowing plants to adapt to changing environmental conditions. In this study, we have identified ECM fungal communities that are associated with white spruce across a strong environmental gradient (nursery → natural forest → forest edge → mining site) near the Sigma-Lamaque mine in the Abibiti region of Quebec, Canada. We hypothesised that white spruce ECM fungal community and diversity differs among sites. We also predicted that soil fertility gradient may play an important role in determining ECM fungal species communities associated with white spruce.

## Materials and Methods

### Ethics Statement

Permit to collect fine roots of white spruce seedlings for each location of the four sites in this study was issued by the appropriate authority. Mr. Michel Miron, Director of Environmental Affairs granted the permission to collect root samples from the natural forest, forest edge and mining site belonging to Sigma-Lamaque Milling Complex and Mine property in Val-d'Or. Mr Marc Castonguay, Director of operations at the Quebec Ministry of Natural Resources, Wildlife and Parks granted the permission to collect root samples from the Trecesson nursery. This root sampling was considered non-disruptive with no negative impact on the environment.

### Study area

The Sigma-Lamaque mine is an open-pit gold mine situated within the municipality of Val d’Or, Quebec. It is located in the balsam fir–white birch bioclimatic zone [39]. This zone occupies the southern part of the Canadian boreal forest. Mature forest stands are mainly composed of balsam fir, white spruce, and white birch on mesic sites [39]. Black spruce, tamarack or eastern larch (*Larix laricina* [Du Roi] K.Koch), trembling aspen, and jack pine dominate less favourable hydric or xeric sites [39]. Forest dynamics are controlled by two types of natural disturbances: (1) outbreaks of eastern spruce budworm (*Choristoneura fumiferana*), which feeds principally on highly abundant balsam fir; and (2) forest fires, which favour the formation of pure stands of jack pine [39].

### Sampling of ECM fungal communities

Four sites near Sigma-Lamaque mine were sampled for their white spruce ectomycorrhizal fungal communities. The Trecesson nursery, TN (Lat. N 48°34'14.43'' and Long. W 78°15'18.78'')), which was located in Amos, 100 km from the mining site, represented the first site. This nursery furnishes most of the tree seedlings that are planted after timber harvesting in the Abitibi region. The mining site, MS (Lat. N 48°06'10.21'' and Long. W 77°45'44.23'') was represented by waste rock piles and fine tailings, which will be restored and revegetated following mine closure. The third site was represented by the forest edge, FE (Lat. N 48°06'48.92'' and Long. W 77°43'39.40'') surrounding the mining site. The fourth site was a natural forest stand, NF (Lat. N 48°05'46.90'' and Long. W 77°42'51.08'') next to Sigma-Lamaque mine. Within each site, five specific plots were randomly selected on a map and geographic coordinates were recorded on a GPS Garmin 60CSx (Garmin International Inc., Olathe, KS, USA). In the field, fine roots of two white spruce trees that were spaced at least 5 m apart were sampled in each location for a total of 10 trees sampled per site following ECM sampling techniques described by Gagné *et al*. [4]. Fine roots were carefully collected in two opposite directions (north–south), starting at the tree base. Roots with surrounding bulk soil were stored in plastic bags at 4°C for up to six days.

### Bulk soil analyses

Three bulk samples of rhizosphere soil were randomly selected in each of the four sites for chemical analyses. Before analyses, soil samples were sieved (2 mm mesh), finely ground, and oven-dried at 65°C for 48 h. Total organic C was quantified following the dichromate oxidation method developed by Yeomans and Bremner [40]. Total N was determined by micro-Kjeldahl digestion procedure with a Lachat flow-injection analyzer [41]. Available soil P was quantified following Bray 2 extraction [42]. Exchangeable K, Ca, Mg, Na, Fe, Mn, and Al were measured by Inductively Coupled Plasma Mass Spectrometer, following techniques developed by Arnacher *et al*. [43]. Finally, pH was measured in a saturated paste extract while electrical conductivity was determined in 1:2 water solution.

### ECM fungal identification

Fine roots were washed gently with tap water over a 2 mm mesh sieve at Université Laval. Roots were stored in tap water in a 150-ml plastic tube until further handling. One hundred ECM root tips of each sample were randomly chosen and characterised based on their colour, texture, form, and size and presence of hyphae and rhizomorphs under a stereomicroscope following the morphotyping techniques used by Wurzburger *et al*. [44]. Mycorrhization percentage for each morphotype was calculated by comparing the number of root tips represented by a specific morphotype with the total number of root tips. Five ECM root tips of each morphotype were then selected, stored in 1.5 mL tubes containing 1% CTAB solution, and maintained at -20°C in a freezer, as described by Quoreshi *et al*. [9], until further molecular analyses could be performed.

Fungal DNA extraction, PCR amplification, and DNA sequencing were performed following the method employed by Gagné *et al*. [4]. Total genomic DNA was extracted using a DNeasy® plant mini kit (Qiagen, Mississauga, ON). The internal transcribed spacer (ITS) of the nuclear ribosomal DNA (rDNA) was amplified using the primers ITS-1F and ITS-4. PCR amplifications were carried out using a PTS-225 thermo-cycler (MJ Research, Waltham, MA). PCR amplifications were accomplished through initial denaturation at 95°C for 2.5 minutes, 30 cycles of denaturation at 95°C for 30 seconds, DNA extension at 72°C for 3 minutes, and a final extension at 72°C for 10 minutes. The products were viewed on 2% agarose gels with 0.5% SynergelTM (Diversified Biotech, Boston, MA) stained with ethidium bromide. For DNA sequencing, a partial ITS sequence for each type of RFLP pattern was determined. Using the amplification primers ITS-1F and a sequenase GC-rich kit (Applied Biosystems, Cleveland, OH), direct sequencing of forward DNA strands was conducted with a dideoxynucleotide chain termination procedure. Sequencing was performed using the ABI 3100 genetic analyser (PE Applied Biosystems, Foster City, CA). Bioedit v7.2.5 software (Ibis Biosciences, Carlsbad, CA) was used to edit raw sequences. Finally, sequences were submitted to BLASTn against the GenBank (http://www.ncbi.nlm.nih.gov) search engine in order to identify ECM fungal taxa to species (≥ 98% homology), genus or family (< 98% homology).

### Numerical analyses

Phylogenetic analyses were performed using Mega 6.0 software developed by Tamura *et al*. [45]. Phylogenetic trees were constructed using maximum parsimony and bootstraping. GenBank taxa that showed the most similar BLASTn results and which had been utilised for ECM fungal identification were included in the first phylogenetic tree. All of our ECM fungal sequences were submitted to the GenBank search engine for public consultation. Genetic distance represented the genetic divergence among species and was calculated for each site. Smaller genetic distances meant that individuals in the same population had more similar genes and were more closely related genetically compared to greater genetic distances.

For each of the four sites, ECM fungal species richness was measured by counting the number of species that were found in the community. Shannon and Simpson’s diversity indices were calculated using species relative abundance data following the method used by Wright *et al*. [46]. Species relative abundance was established as the number of root tips colonized by each fungal species divided by the total number of root tips that were sampled per site, multiplied by 100. Species relative frequency was calculated for each sampling site by dividing the number of samples in which a species occurred by the total number of samples. Mean percentage of roots that were colonised by ECM fungi (± standard error) was calculated for each site by averaging the percentage of ECM root tips from the 10 tree samples. No data transformation was necessary in order to meet ANOVA assumptions. Significantly different means at *P*-value ≤ 0.05 were determined based on one-way ANOVA followed by Tukey tests [47].

All multivariate analyses were executed with PC-ORD 6 (MJM Software Design, Gleneden Beach, OR) [48]. A PerMANOVA (permutational MANOVA) analysis including pairwise comparisons was performed to determine whether any of the sampling sites differ significantly in terms of their ECM fungal species composition. Additionally, two-way cluster analysis was performed to identify which groups of ECM fungi were abundant in same habitat and which sites had similar species compositions. Species relative abundance data served as the starting point for these analyses. Species with horizontal line above the 75% of information remaining are considered to be significantly in the same ecological group in PC-ORD 6, as recommended by Peck [49]. In order to recognize similar linear patterns among soil chemical properties (pH, % N, % C, C/N, P, Ca, K, Mg, Fe, Na, Mn, and AlOH) and mycorhization variables across the ecological gradient, principal component analysis (PCA) was subsequently conducted on the four sampling sites in PC-ORD 6, as recommended by Peck [49]. The significance of differences and linear patterns was set at *P* ≤ 0.05 for all multivariate analyses.

## Results

### ECM fungal community and phylogenetic trees

**Fig 1:**
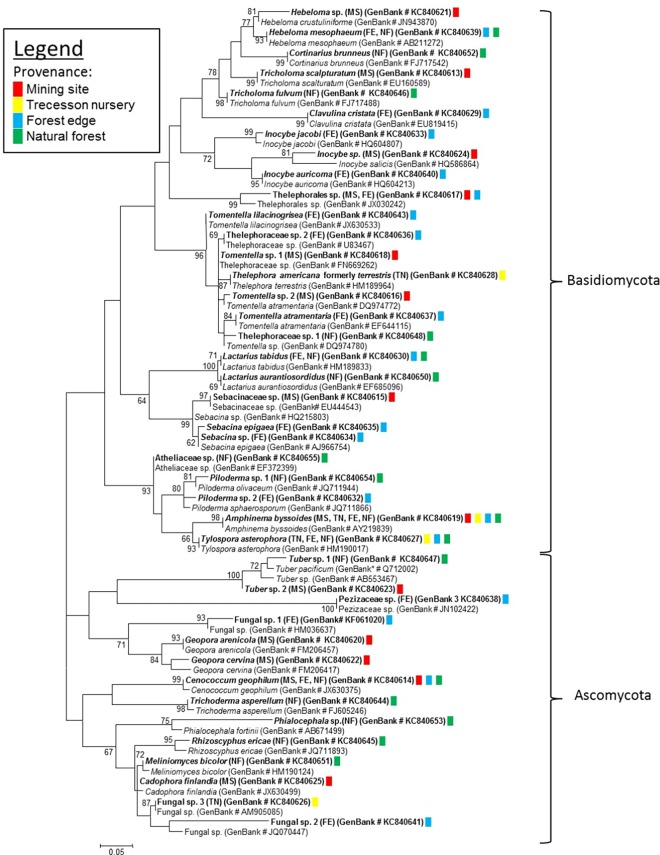
Phylogenetic tree containing all ECM fungal taxa (in bold) that have been identified on the four sites (mining site (MS) in red, Trecesson nursery (TN) in yellow, forest edge (FE) in turquoise, and natural forest (NF) in green) with their corresponding GenBank accession number–Taxa not in bold refer to the most similar blastn results and GenBank taxa used for ECM fungal species identification.

**Fig 2:**
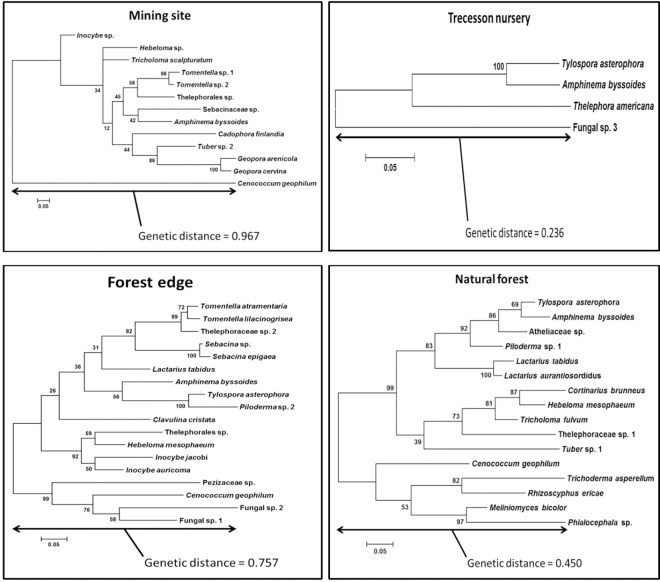
Phylogenetic trees showing the ECM fungal community of each site and the genetic distance among species within site.

In total, 111 morphotypes were described. Through molecular analyses, 90 of them (81.1%) were successfully identified, revealing a total of 41 different ECM fungal species that were associated with white spruce tree roots, including 27 members of the Basidiomycota and 14 Ascomycota (Fig 1). The main phylogenetic tree shows the evolutionary relationships among the 41 identified ECM fungal species (shown in bold with their respective GenBank numbers) (Fig 1). Species within each of the four sampling sites are well distributed throughout the phylogenetic tree (see coloured squares in Fig 1). Genetic distance among ECM fungal species was the highest for the mining site (0.967) followed by forest edge (0.757), natural forest (0.45), and lastly, the Trecesson nursery (0.236) (Fig 2). Therefore, genetic similarities among species across the soil fertility gradient were at the lowest for the mining site, increasing from the forest edge to the natural forest, and highest for the nursery.

### Species abundance, frequency, and diversity

**Fig 3:**
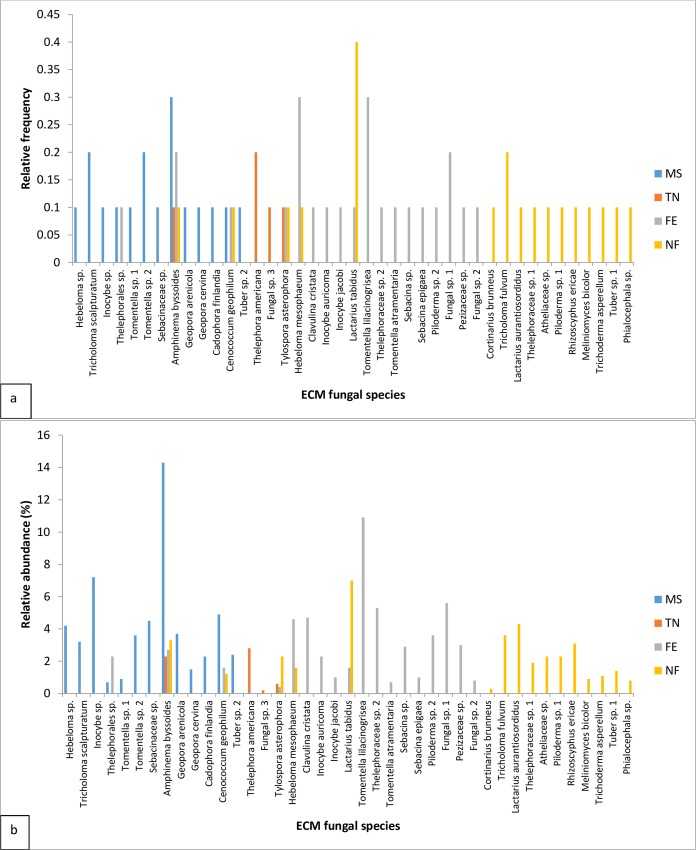
(a) Relative frequency and (b) Relative abundance of all identified ECM fungal species within each sampling site (mining site (MS), Trecesson nursery (TN), forest edge (FE), and natural forest (NF)).

*Amphinema byssoides* P. Karst (Atheliaceae) was the most frequent ECM species associated with white spruce at the mining site, followed by *Tricholoma scalpturatum* (Fr.) Quél. (Tricholomataceae) and *Tomentella* sp.2 (Fig 3A). *Thelephora americana* Peck Sacc. (Thelophoraceae) was the most common species found on white spruce roots in the nursery (Fig 3A). Along forest edges, *Hebeloma mesophaeum* (Pers.) Quél. (Hymenogastraceae) and *Tomentella lilacinogrisea* Wakef. (Thelophoraceae) were the two most frequent species, followed by *Amphinema byssoides* and Fungal sp. 1 (Fig 3A). Finally, *Lactarius tabidus* (Fr.) (Russulaceae) was the most common ECM fungal species found on white spruce roots in the natural forest, followed by *Tricholoma fulvum* (Fr.) Bigeard & Guill. (Tricholomataceae) (Fig 3A). All other species occurred at low frequency (0.1) within their corresponding sites (Fig 3A).

Further, *A*. *byssoides* was the most abundant (14.3%) ECM species associated with white spruce on the mining site (Fig 3B). The second most abundant species was *Inocybe* sp. (7.2%) and all of other species tended to have relative abundances less than 5% (Fig 3B). At the nursery, *T*. *americana* (2.8%) and *A*. *byssoides* (2.3%) had the highest relative abundances (Fig 3B); all other species had very low abundances, viz., less than 1% (Fig 3B). At the forest edge, *T*. *lilacinogrisea* was the most abundant species (10.9%), followed by *H*. *mesophaeum*, *Clavulina cristata* (Holmsk.) J.Schröt. (Clavulinaceae), Thelephoraceae sp. 2, and Fungal sp. 1, all of which has relative abundances of about 5% (Fig 3B). The remaining species on this site all had relative abundances less than 4% (Fig 3B). In the natural forest, the most abundant species was *L*. *tabidus* (7%), followed by *T*. *fulvum* and *Lactarius aurantiosordidus* (each with about 4%) (Fig 3B). All remaining species on this site had relative abundances less than 4% (Fig 3B).

Species richness among the sites was 13, 18, 16, and 4, corresponding to the following soil fertility gradient: mining site → forest edge → natural forest → nursery. Associated Shannon diversity indices (H’) were respectively 2.29, 2.61, 2.55, and 1.07. Simpson’s diversity (D) was 0.87, 0.91, 0.91, and 0.61, respectively. Trecesson nursery had a significantly much lower percentage of white spruce roots colonised by ECM fungi (6.9 ± 2.3) than did the natural forest (56.8% ± 4.7%), forest edge (63.7% ± 4.8%) and mining site (69.2% ± 1.9%).

### Shaping groups with similarities

**Fig 4:**
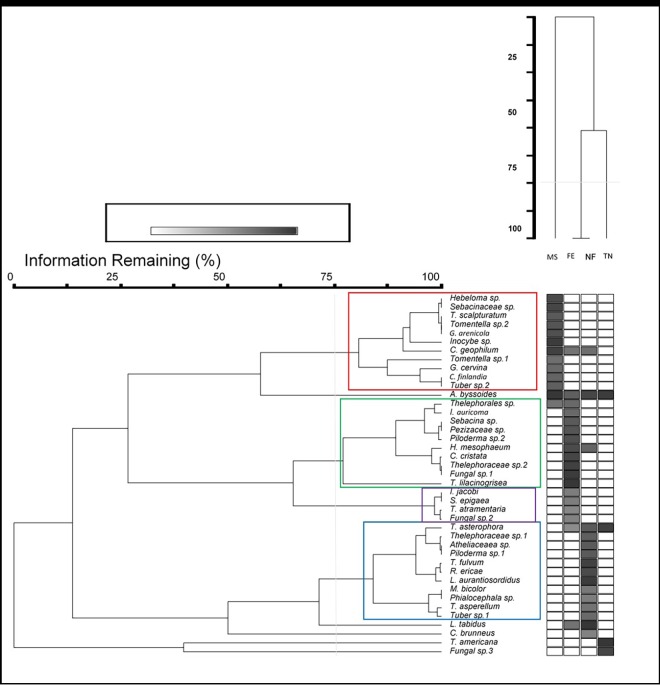
Two-way cluster analysis (α = 0.05) showing groups of species thriving in same habitats (small coloured rectangles) (darker grey squares mean higher abundance; Big red rectangle = no difference between site species composition).

ECM fungal communities differed between at least two of the four sampling sites (*P =* 0.0004), according to the PerMANOVA. We further demonstrated that fungal communities significantly differed among all sites (PerMANOVA adjusted *P*-values: *P* = 0.0154, MS vs TN; *P* = 0.0424, MS vs FE; *P* = 0.0150, MS vs NF; *P* = 0.0082, TN vs FE; *P* = 0.0056, TN vs NF), except between forest edge and natural forest (adjusted *P*-value = 0.1204). Two-way cluster analysis further confirmed these findings. According to this analysis, fungal species communities of the forest edge and natural forest were significantly similar (> 75% information remaining), while species compositions of the other sites were different (< 75% information remaining) (Fig 4).

In the two-way cluster dendrogram, four different groups of ECM fungal species could be recognised to persist together in similar habitats (> 75% information remaining) (Fig 4). In this study, *Hebeloma* sp., Sebacinaceae sp., *Tricholoma scalpturatum*, *Tomentella* sp. 2, *Geopora arenicola* (Lév.) Kers (Pyronemataceae), *Inocybe* sp., *Cenococcum geophilum* Fr. (Gloniaceae), *Tomentella* sp. 1, *Geopora cervina* (Velen.) T.Schumach.(Pyronemataceae), *Cadophora finlandia* (C.J.K. Wang & H.E. Wilcox) T.C. Harr. & McNew (Heliotiaceae), and *Tuber* sp. 2 were a group of ECM fungal species that colonised white spruce roots on the mining site (Fig 4). Thelephorales sp., *Inocybe auricoma* (Batsch) J.E.Lange (Inocybaceae), *Sebacina* sp., Pezizaceae sp., *Piloderma* sp. 2, *H*. *mesophaeum*, *C*. *cristata*, Thelephoraceae sp. 2, Fungal sp. 1, and *T*. *lilacinogrisea* were found together in white spruce rhizospheres in the forest edge site (Fig 4). A second group of species composed of *Inocybe jacobi* Kühner (Inocybaceae), *Sebacina epigaea* (Berk. & Broome) Neuhoff (Sebacinaceae), *Tomentella atramentaria* Rostr. (Thelephoraceae), and Fungal sp. 2 colonised white spruce roots, in lower abundance, in the forest edge site (Fig 4). Last, the fourth group of ECM fungal species which included *Tylospora asterophora* (Bon.) Donk (Corticiaceae), Thelephoraceae sp. 1, Atheliaceae sp., *Piloderma* sp. 1, *T*. *fulvum*, the ericoid *Rhizocyphus ericae* (D.J. Read) W.Y. Zhuang & Korf (Helotiaceae), *Lactarius aurantiosordidus* Nuytinck & S.L. Mill. (Russulaceae), *Meliniomyces bicolor* Hambl. & Sigler (Heliotiaceae), *Phialocephala* sp., *Trichoderma asperellum* Samuels, Lieckf. & Nirenberg (Hypocreaceae), and *Tuber* sp. 1 formed a closely related community within the white spruce rhizosphere in the natural forest (Fig 4). *Amphinema byssoides* was encountered in all four sampling sites (Fig 4). Nevertheless, this ECM fungal species was more closely associated with white spruce seedlings naturally regenerating on the mining site (Fig 4). *Lactarius tabidus* and *Cortinarius brunneus* var. brunneus. (Pers.) Fr. (Cortinariaceae) were more closely associated with the natural forest, but they did not form any closely related groups (Fig 4). *Thelephora americana* and Fungal sp. 3 were important ECM fungal species that inhabited the rhizosphere of white spruce seedlings at the Trecesson nursery (Fig 4).

### Looking for linear patterns

**Fig 5:**
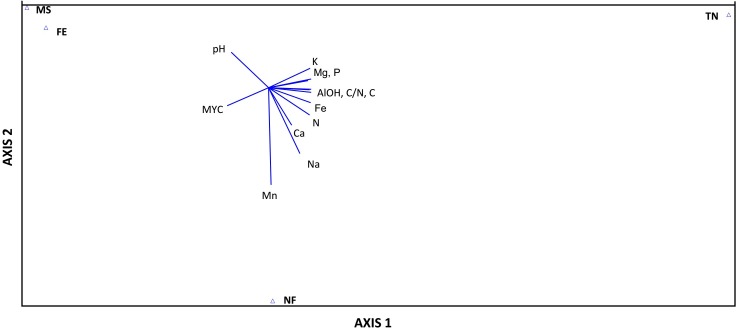
Principal component analysis (α = 0.05) showing linear patterns of a wide range of variables across the ecological gradient (Sampling sites: MS = mining site, FE = forest edge, NF = naturel forest, and TN = Trecesson nursery) (only the axis 1 is significant; closer to center means smaller values) (MYC = % of roots colonized by ECM fungi).

Principal component analysis (PCA) allowed us to identify linear patterns within soil chemical and mycorrhization data across the soil fertility gradient. Axis 1 explained 78.6% of variation in the data with a very small *P*-value (0.0010) reflected by the randomisation tests. Therefore, the Axis 1 significantly showed linear patterns (Fig 5). In contrast, Axis 2 and 3 were not significant for linear patterns with *P-*values of 1.0000 and 0.9990, respectively. Thus, they were not further considered in this multivariate analysis. Recommendations from the different stopping rules did not considerably vary, so the assumption of data homogeneity was met. In examining the PCA ordination of the mining site, forest edge, natural forest and Trecesson nursery, soil pH and the percentage of root tips colonised by ECM fungi decreased across the gradient formed by Axis 1, while soil K, Mg, P, N, C, Fe, Ca, Na, and AlOH content and C/N ratio tended to increase (Fig 5).

## Discussion

### Genetic divergence within communities

ECM fungal species that were located in each of the four sites were well distributed across the whole phylogenetic tree. Genetic divergence among species was at its highest on the mining site. These results were quite surprising. One would think that genetic divergence among species for the mining site, compared to other sites, would be considerably low given that only a small group of fungi (order, family or genus) would have successfully adapted to harsh conditions. However, it is not the case here. ECM fungal adaptation to the mining site may be strongly controlled by high genetic variability within and among species, which allows some ectomycorrhizal fungal ecotypes that are adapted to site conditions to colonise highly disturbed and contaminated ecosystems [50]. This hypothesis agrees with results that were obtained by Colpaert *et al*. [27] and Adriaensen *et al*. [51], where tolerance of the ECM fungus *Suillus luteus* (L.:Fries) Gray (Suillaceae) to soils with high concentrations of Zn, Cd, and Cu was much higher for strains that had been isolated from metal-polluted habitats than those isolated from non-polluted soils. Colpaert [52] reviewed the ecological and evolutionary processes that drive plant and fungal communities and populations on metal-contaminated sites. He reported that heavy metal toxicity is a strong trigger for evolutionary and genetic adaptations in ectomycorrhizal fungi that colonise metalliferous soils.

### ECM fungal species diversity

*Amphinema byssoides* was the most frequent and abundant ECM fungal species colonising white spruce roots on the mining site. Hunt [53] also suggested that *A*. *byssoides* is an early successional fungal species. Furthermore, this species was found on all sites. *A*. *byssoides* may be a generalist species that thrives in a wide range of habitats. *Tricholoma scalpturatum* and *Inocybe* sp. had a relatively high frequency and abundance, respectively, on the mining site. *T*. *scalpturatum* has also been known for colonising roots of European aspen (*Populus tremula* L.) on mining sites that are contaminated by lead, zinc, and cadmium [54]. *Thelephora americana* was the most frequent and abundant ECM fungal species colonising white spruce seedlings at the nursery. Kernaghan *et al*. [5] and Quoreshi *et al*. [9] also found *T*. *americana* colonising white spruce in nurseries. This species may be well adapted to high fertilization regimes. *Hebeloma mesophaeum* and *Tomentella lilacinogrisea* were the two most frequent and abundant species colonising white spruce roots at the forest edge. As far as we know, this is the first study that has looked at ECM fungal communities in forest edge ecotone. *Lactarius tabidus* was the most frequent and abundant ECM fungal species colonising white spruce roots in the natural forest. Wright *et al*. [46] found similar results with two different species within the genus *Lactarius*, which were among the top five most frequent and abundant ECM species on western hemlock (*Tsuga heterophylla* [Raf.] Sarg.) roots in natural forest stands.

The number of species that were associated with white spruce seedlings was much smaller in the nursery compared to the other three sites. Seedlings in the nursery were amended regularly with fertilisers containing high concentrations of N. Lilleskov *et al*. [55] found that ECM fungal species richness tremendously decreased on white spruce roots with increasing soil N content. As many as 30 taxa were identified on sites with low N content, while only nine were encountered in high N content sites [55]. Nilsson & Wallander [56] and Lilleskov *et al*. [57] also demonstrated that N fertilisation considerably reduces ECM fungal species richness. Overall, species diversity was increasing from the nursery, the mining site, the natural forest to the forest edge. ECM fungal diversity for conifers is usually lower in early successional ecosystems compared to late-successional forest stands [58], which agrees with our diversity results.

### Difference in species composition among sites

In this study, we found no difference in ECM fungal species composition on white spruce roots between the forest edge and natural forest. In contrast, the species composition of trees from the Trecesson nursery differed from those of the forest edge and natural forest. The greatest difference in fungal species composition associated with white spruce was between the mining site and the three other sites: nursery, forest edge, and natural forest. These findings agree with Twieg *et al*. [58], who showed that ECM fungal species compositions were similar in older forest stands but differed when young stands were compared to old ones. Stand age seems to explain a large part of the variation that is observed among ECM fungal communities [58]. Wright *et al*. [46] demonstrated that ECM fungal species composition differed considerably between N-P-fertilised trees and those that were left unfertilised. Therefore, fertilisation is likely the reason why species composition at the nursery was unlike the other three sites. Yet, what could explain the difference in species composition between the mining site and the surrounding forest edge and natural forest? In a study aimed at examining relationships among ECM fungal community measures, local soil nutrients, and stand age along a chronosequence of mixed forest stands in Interior Cedar-Hemlock forests of southern British Columbia, Twieg *et al*. [59] found that ECM fungal community structure was more strongly influenced by stand age than specific soil nutrients, but better correlations with soil nutrients can occur at broader spatial scales with wider range of site qualities.

After major disturbance events such as mining, soil microbial community is depauperate [60]. In these primary successional ecosystems, the vast majority of ECM fungal propagules colonise the newly-disturbed ecosystem through aerial spore dispersal and transport, which allows establishment of a dormant spore bank in these rocky soils [60]. Every ECM fungal species selects its own optimal set of environmental conditions in which it can grow more effectively [60]. Furthermore, niches vary greatly among ECM fungal species [60]. Species that were colonising the mining site are probably present in the natural forest in low abundance and frequency because site conditions are not optimal for them. At one point, spores may be transported by wind to the mine tailings. Environmental pressures of the mining site allow the selection of the most adapted species and strains, which later colonise newly regenerating seedlings [60]. Colpaert *et al*. [50] have shown the genetic variability of ectomycorrhizal fungal ecotypes that are adapted to site conditions to colonise highly disturbed and contaminated sites. This colonisation hypothesis is the most likely reason why the ECM fungal community that is associated with white spruce on the mining site is significantly different from communities of the surrounding environments.

### ECM fungal communities and soil chemical properties

In this study, soil exchangeable K, Mg, P, Fe, Ca, Na and Al concentrations, C/N ratio, and N and C contents decreased when moving from the nursery, natural forest and forest edge to the mining site, while pH and the percentage of white spruce roots that were colonised by ECM fungi increased along the soil fertility gradient. The first group of ECM fungi inhabiting the roots of white spruce trees on the mining site were adapted to low soil fertility, very low organic matter content (OMC—low C content), and high pH. The second and third groups (forest edge) were adapted to similar but not as low soil fertility, high pH, and low OMC conditions than those present on the mining site. Here, we suspect that the forest edge and the mining site had relatively similar soil chemical properties due to transport of tailing fine particles by wind erosion from the mining site to the surrounding forest edge, causing a change in soil chemical properties at the soil surface, which would normally be much more similar to natural forests. The fourth group of ECM fungi (natural forest), in contrast, were adapted to higher soil fertility, higher OMC, and lower pH. As a result, soil chemical properties played an important role in ECM fungal species composition of the four sampling sites. Toljander *et al*. [21] also found that ECM fungal community structure is strongly related to soil properties such as extractable NH_4_^+^, base saturation, pH, and C/N ratio. Furthermore, Bahram *et al*. [61] demonstrated that soil nutrient content was one of the key factors controlling ECM community composition. Other studies also have shown a strong relationship between soil edaphic characteristics and ECM fungal community composition [38,62,63]. Kennedy [64] suggested that competition among species considerably affects the interactions between them and has a large influence on community structure. ECM fungal competitive efficiency is dependent upon environmental conditions such as soil pH, temperature, and nutrient content [64]. As a result, species with the highest abundance and frequency may be better adapted to site conditions, thereby enabling them to outcompete others.

In this study, the percentage of white spruce roots that were colonised by ECM fungi increased as soil chemical fertility and organic matter content decreased and as pH rose. Total fungal biomass tends to be higher in soils with low nutrient availability and low tree productivity [65]. In the past, field trials have clearly demonstrated the importance of mycorrhizal colonisation on the survival and growth of tree seedlings in primary successional ecosystems [60]. It may explain why the roots of healthy naturally regenerating white spruce seedlings on the mining site had such high ECM fungal colonisation rates.

### Edaphic selection pressures–drivers of ECM fungal community structure

It is well known that ECM fungi are, in general, acidophilic symbiotic microorganisms that preferentially thrive in poor acid soils with highly recalcitrant litter content [21]. However, in this study, we identified few ECM fungi that were ecologically adapted to poor alkaline soils with low organic matter content. These microsymbionts successfully colonised the root system of young white spruce seedlings on the non-acidogenic mine tailings of Sigma-Lamaque mine, allowing scarce natural plant regeneration. Once isolated, they are potential bioinoculants for use in enhanced ecorehabilitation of Precambrian gold mining rocks of the Canadian shield [30]. In contrast, other ECM fungi are adapted to rich acid soils of the commercial nursery. Edaphic selection pressures after fungal spore dispersal lead to the selection of the most site-adapted ECM fungi through species survival and competition dynamics [38,60]. As a result, edaphic selection pressures are important factors controlling ECM fungal community structure, composition, and diversity. There is a symbiotic modulation that occurs both temporally and spatially in the disturbed versus natural environment allowing plants to adapt to changing environmental conditions.

## Conclusion

ECM fungal community composition of the mining site was significantly different compared to the surrounding environments: Trecesson nursery, forest edge, and natural forest. To survive on white spruce roots on the mining site, ECM fungi had to be adapted to high pH, and low soil fertility and organic matter content. ECM fungal adaptation to the mining site seems to be more related to high genetic variability within and among species than phylogenetic group adaptation. White spruce seedlings that were naturally regenerating on the mining site were heavily colonised by ECM fungi. This study clearly shows the contrasting difference in white spruce ectomycorrhizal fungal diversity and community structure that is driven by edaphic selection pressures.
